# E3 Ubiquitin ligase NEDD4 family‑regulatory network in cardiovascular disease

**DOI:** 10.7150/ijbs.48437

**Published:** 2020-08-21

**Authors:** Ying Zhang, Hao Qian, Boquan Wu, Shilong You, Shaojun Wu, Saien Lu, Pingyuan Wang, Liu Cao, Naijin Zhang, Yingxian Sun

**Affiliations:** 1 Department of Cardiology, the First Hospital of China Medical University, Shenyang, Liaoning, P.R. China.; 2 Staff scientist, Center for Molecular Medicine National Heart Lung and Blood Institute, National Institutes of Health, the United States.; 3 Key Laboratory of Medical Cell Biology, Ministry of Education; Institute of Translational Medicine, China Medical University; Liaoning Province Collaborative Innovation Center of Aging Related Disease Diagnosis and Treatment and Prevention, Shenyang, Liaoning, China.

**Keywords:** NEDD4 E3 ligases, post translation modification, ubiquitin proteasome system, cardiovascular disease

## Abstract

Protein ubiquitination represents a critical modification occurring after translation. E3 ligase catalyzes the covalent binding of ubiquitin to the protein substrate, which could be degraded. Ubiquitination as an important protein post-translational modification is closely related to cardiovascular disease. The NEDD4 family, belonging to HECT class of E3 ubiquitin ligases can recognize different substrate proteins, including PTEN, ENaC, Nav1.5, SMAD2, PARP1, Septin4, ALK1, SERCA2a, TGFβR3 and so on, via the WW domain to catalyze ubiquitination, thus participating in multiple cardiovascular-related disease such as hypertension, arrhythmia, myocardial infarction, heart failure, cardiotoxicity, cardiac hypertrophy, myocardial fibrosis, cardiac remodeling, atherosclerosis, pulmonary hypertension and heart valve disease. However, there is currently no review comprehensively clarifying the important role of NEDD4 family proteins in the cardiovascular system. Therefore, the present review summarized recent studies about NEDD4 family members in cardiovascular disease, providing novel insights into the prevention and treatment of cardiovascular disease. In addition, assessing transgenic animals and performing gene silencing would further identify the ubiquitination targets of NEDD4. NEDD4 quantification in clinical samples would also constitute an important method for determining NEDD4 significance in cardiovascular disease.

## Introduction

Protein ubiquitination, a critical post translation modification, is necessary for ubiquitin-associated protein degradation by the ubiquitin proteasome system (UPS). UPS system comprise ubiquitin activating enzyme (E1), ubiquitin conjugating enzyme (E2), and ubiquitin ligase (E3). First, ubiquitin activation is performed by E1. Then, E2 transfers ubiquitin to E3. Finally, E3 catalyses the covalent binding of ubiquitin to the target protein 1. E3 ligases are divided into three main families: zinc-binding RING finger adaptor (a recently discovered gene) 2, HECT (homologous with the carboxyl end of E6AP) catalytic 3 and U-box (a modified cyclic motif) families 4. E3 enzymes involve in substrate recognition and ubiquitin transfer to a single, multiple Lys (mono-/multi-ubiquitination) or as a poly-Ub chain (poly-ubiquitination) residues of the substrate (HECT family) 5 or easing ubiquitin transfer from E2 to the target protein (RING family). All seven lysine (K6, K11, K27, K29, K33, K48 and K63) and the N-terminal M1 residues constitute linkage points during chain elongation 6, although lys48 and lys63 are mostly involved. K48-, K11-, K29 linked poly-Ub chains direct substrates to proteasomal degradation 7, 8, while mono-ubiquitination and K63-linked poly-Ub chains have non-proteolytic functions 9. In contrast, K63-linked chains control “proteasome-independent” events, including inflammation-related signaling pathways, DNA repair, endocytosis, and selective autophagy 10,11. Nedd4-family E3 members mostly synthesize K63-bound poly-Ub chains 12, 13, unlike other HECT ligases. This is followed by protein recognition and degradation by the 26S proteasome 5, 14. The neuronal precursor cell-expressed developmentally downregulated 4 (NEDD4) family constitutes an important group in the HECT group 15, 16.

**Figure 1:**
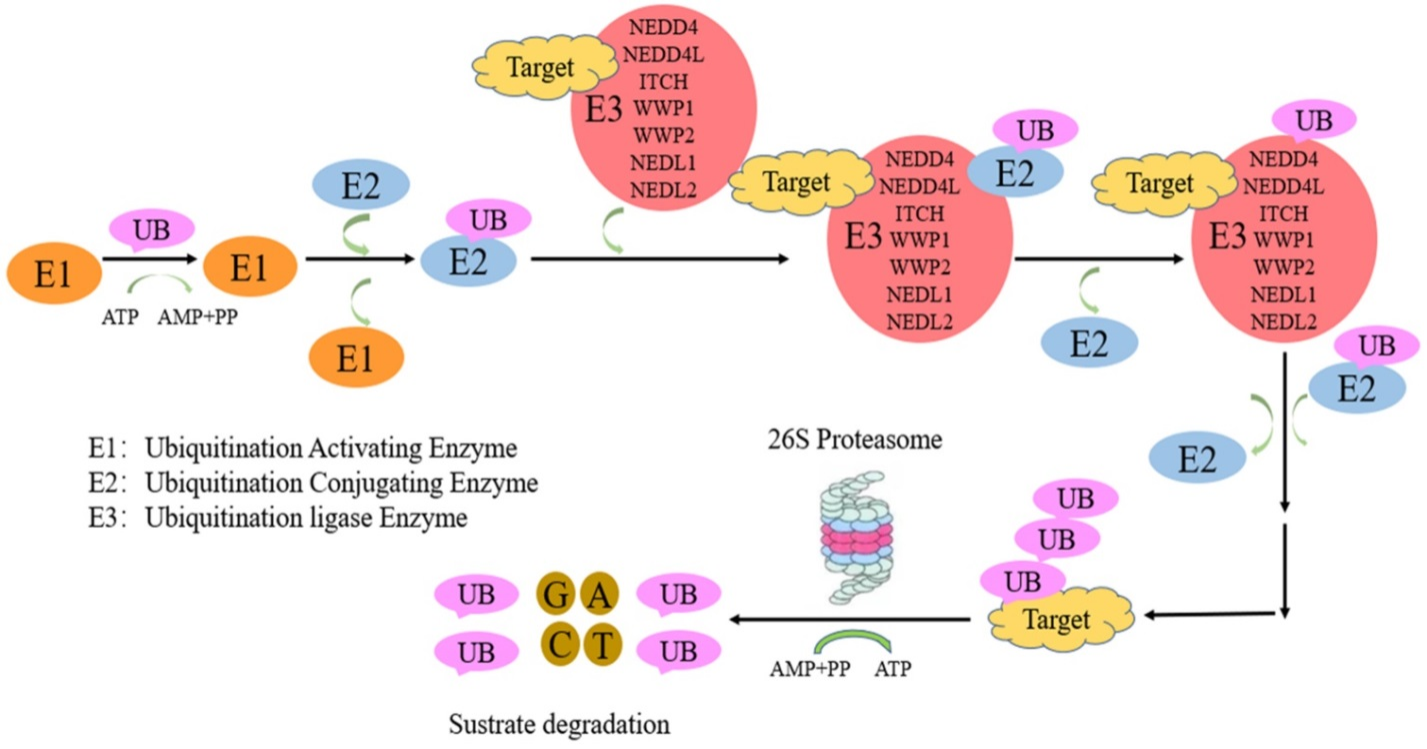
** The ubiquitination-proteasome of NEDD4 E3 ligases pathway.** Proteins targeted for UPS-induced degradation by NEDD4 E3 ligases comprise polyubiquitin chain by a process involving three proteins, including ubiquitin activating enzyme (E1), ubiquitin conjugating enzyme (E2), and ubiquitin ligase (E3). First, ubiquitin activation is performed by E1. Then, E2 transfers ubiquitin to E3. Finally, NEDD4 E3 ligases catalyses the covalent binding of ubiquitin to the target protein.

NEDD4-1 and NEDD4-like (NEDD4L or NEDD4-2) enzymes represent E3 ubiquitin-protein ligases of the HECT family 17, 18. NEDD4 family enzymes are conserved from yeast to humans 19. As shown in **Figure 1,** the NEDD4 family contains nine members in humans: NEDD4-1 (RPF1), NEDD4L (NEDD4-2), ITCH/atropine-1 interaction protein 4 (AIP4), WW domain-containing E3 ubiquitin protein ligase 1 (WWP1), WWP2/Atropine-1-interacting protein 2 (AIP2), NEDL1 (HECW1), NEDL2 (HECW2), SMAD-specific E3 ubiquitin protein ligase 1 (SMURF1) and SMURF2 20. NEDD4 family enzymes structure consists of N-terminal C2 domain (Ca^2+^/phosphorlipase and membrane-binding; mediating Ca^2+^-associated targeting to the cell membrane substrate recognition 21), 2-4 WW domain and catalytic C-terminal domain (HECT; 350 residues controlling ubiquitin binding to e-NH2 groups of lysine residues on protein substrates 5, 18, 22). The WW domain catalyzes protein ubiquitination and catabolism (or endocytosis) 15. The WW domain recognizes and binds to predominantly proline-rich sequences on substrate proteins 23, e.g., PPxY (where x represents any amino acid) 24, PPLP 25, PR 26 and phosphoserine/threonine (pS/pT) residues preceding proline 27. The catalytic HECT domain consists of an "n-leaf" and a catalytic cysteine residue in a "c-leaf" which confers the ligase its catalytic activity 28. NEDD4 E3 ligases differ in function because of distinct WW domains 29 and different substrates 16. In the past few years, the ubiquitin proteasome system has been attributed key roles in regulating diverse cardiovascular disease 30, 31, including atherosclerosis, familial cardiopathy, idiopathic dilated cardiomyopathy and myocardial ischemia 32. Here we review the current evidence accumulated concerning the cardiovascular role of NEDD4 family members in cardiovascular disease.

## NEDD4-1

### NEDD4-1 function

NEDD4-1 was first isolated from mouse neural progenitor cells in 1992, with reduced mRNA levels during mouse brain development. *NEDD4-1* is widely expressed in the heart, lung, brain, somite, kidney, and other tissues. It may be involved in many human cell functions 33. NEDD4-1 catalyses the degradation of its substrate by polyubiquitination at K48 and K63 34-36 or by single ubiquitination of K6 or K27 37-38, indicating that NEDD4-1 plays a variety of regulatory roles through single/multiple ubiquitination.

### The role of NEDD4-1 in myocardial reperfusion injury

Myocardial reperfusion injury involves tissue damage occurring with blood supply return to the cardiac tissue following ischemia or lack of oxygen, causing inflammation 39. NEDD4-1 expression is reduced in the late stage of ischemia/reperfusion (I/R), thereby attenuating its protective effects against cell death and cardiac I/R injury. In addition, activation of the AKT serine/threonine kinase (AKT) pathway protects the heart from I/R injury 40. NEDD4-1 promotes nuclear trafficking of active AKT. Phosphatase and Tensin Homolog (PTEN) represents a critical suppressor of Phosphatidylinositol-3-Kinase (PI3K) signaling and is controlled by NEDD4-1 via polyubiquitination 41-42. PTEN-associated AKT inhibition is suppressed by NEDD4-1, while AKT signaling is activated to protect against I/R-induced cell damage and apoptotic cell death, as demonstrated by decreased BAX and cleaved-caspase3/7 levels and elevated BCL2 amounts. Therefore, NEDD4-1 protects the myocardium from I/R induced apoptosis by activating PI3K/Akt pathway. This regulatory pathway provides a novel insight into reducing the intraoperative injury in patients with myocardial infarction.

### The role of NEDD4-1 in heart development

In embryonic day 10.5 (E10.5) mice, NEDD4-1 is expressed in the pharyngeal and gill arches near the heart. *Nedd4* knockout mice show severe heart and vascular defects in the second of pregnancy, leading to fetal death, accompanied by significant heart defects and vascular system abnormalities. In particular, outflow tract defects in knockout animals display a double outlet right ventricle 43. *Nedd4* knockout mice also have endocardial cushion defects. Abnormalities are also present in the vascular system of *Nedd4* knockout mice. In particular, the cephalic plexus vein in some knockout embryos is abnormal. In addition to these significant cardiovascular defects, *Nedd4* knockout embryos exhibit delayed maturation of the lungs, where *Nedd4* is highly expressed 44.

### The role of NEDD4-1 in Vascular Calcification

Vascular calcification represents a major complication of atherosclerosis, chronic renal failure, diabetes, cardiovascular disease and other pathological conditions. More and more evidences show that vascular calcification likens osteogenesis 45-47. Among TGFβ superfamily proteins, BMP2 and TGFβ1 can be targeted for the treatment of multiple bone disorders. In previous study, NEDD4-1 suppression in SM22α^+^ mouse tissues resulted in deformed aortic structures. Meanwhile, Vitamin D-associated aorta vascular calcification was markedly elevated in *Nedd4*-null animals compared with wild type littermates. In addition, methylation of human NEDD4 gene promoter is remarkably enhanced in individuals with atherosclerosis. Further research indicated that NEDD4-1 E3 ligase is an important BMP/Smad signaling inhibitor, via polyubiquitination-associated degradation of C-terminal phosphorylated Smad1 (pSmad1) activated by TGF-β. Thus, dysregulated or dysfunctional NEDD4-1 E3 ligase could contribute to vascular calcification in VSMCs through induction of bone generating signals in the process of atherosclerosis progression 48.

## NEDD4L

### NEDD4L function

NEDD4L on human chromosome 18q21 contains the WW and HECT domains as similar to other NEDD4 genes. In mice, Nedd4L shows homology with Nedd4-1, but has no C2 domain in the N-terminus region. In addition, human NEDD4L and mouse nedd4-2 are homologous 49,50. Nedd4L is widely expressed during mouse development and in adult tissues, especially in the liver, kidney, heart, brain and lung 51,52. Furthermore, NEDD4L plays a vital role in hypertension and arrhythmia.

### The role of NEDD4L in hypertension

Hypertension represents an important risk factor for cardiovascular ailments, including myocardial infarction, stroke, heart failure and kidney disease. It reflects salt-sensitivity or resistance when blood pressure response to high- or low-salt diets intake varies substantially 53-54. Epithelial sodium channel (ENaC) suppression in the kidney, lung via the NEDD4 family is important in fluid and electrolyte homeostasis. The ENaC significantly affects kidney Na^+^ reabsorption 55. It is a hetero-multimeric membrane protein consisting of the homologous subunits α, β, and γ, each comprising intracellular N and C terminal 55. NEDD4L was originally identified as a ligand of the ENaC; NEDD4L binds to β- and γ-subunits of the ENaC through the ENaC proline-tyrosine (PY) motif in the C-terminal region, interacting with the WW domain in NEDD4L, followed by ubiquitination and degradation 56-58. In Liddle's syndrome, an ENaC incorporating variant β or γ subunits that lack the PY motif leads to deficient interaction with the WW domain of NEDD4L, resulting in excessive Na^+^ reabsorption and hypertension, with the characteristics of salt-sensitivity, hypokalaemia, metabolic alkalosis, and reduced renin activity and aldosterone amounts 22, 59-61. *Nedd4* knockout mice display high blood pressure even with normal diets, an aliment exacerbated with high-salt diets 62. The IL17A-SGK1/NEDD4L-dependent pathway modulates renal sodium transport by improving renal function in hypertension and other autoimmune disorders. Glucocorticoid Regulated Kinase 1 (SGK1) induced by serum and glucocorticoids triggers a cascade that leads to hypertension by ENaC activation 61-62. SGK1 phosphorylation of NEDD4L promotes its interaction with the chaperone 14-3-3, thus eliminates the ubiquitination of its target substrates 63-64. SGK1 phosphorylates NEDD4L on serine 444 65, which is necessary for the binding of NEDD4L to 14-3-3, and results in a reduction of ENaC ubiquitination and an enhancement of ENaC activity mediated by NEDD4L. SGK1 is activated by WNK1 (WNK Lysine Deficient Protein Kinase 1), which is involved in pseudo-hypoaldosteronism type II hypertension. WNK1 activates SGK1, inhibits NEDD4L, enhances ENaC activity and leads to hypertension 66. ENaC activity was also observed during the inhibition of NEDD4L by AMP-activated Protein Kinase (AMPK) 65. These results indicate that IL17A induces an increase in NCC activity through SGK1 phosphorylation and the inhibition of NEDD4L-mediated ubiquitination and NCC degradation. These studies provide a mechanistic link by which the IL17A-SGK1/NEDD4L-dependent pathway modulates renal sodium transport, which may improve renal function in hypertension and other autoimmune disorders 67.

### The role of NEDD4L in arrhythmia

Reduced voltage-gated sodium channel (Nav1.5) function and expression supply a slowed conduction substrate for heart arrhythmias. Calcium-associated increases in NEDD4L reduces Nav1.5 levels by ubiquitination. Nav1.5 co-localizes with NEDD4L, and ubiquitin is downregulated in the failing heart in rats. The above findings indicate an important role for NEDD4L in Nav1.5 suppression in heart failure (HF) 68. In addition, defection of the NEDD4L C2 isoform changes cardiac conduction in the resting state as well as pro-arrhythmic alterations upon acute myocardial infarction (MI). Studies indicated that reduced NEDD4L function results in serious heart arrhythmia via modifications of cardiac ion-channels after transcription. Patients with salt sensitivity and hypertension because of NEDD4L anomalies may be more prone to severe cardiovascular ailments compared with individuals without NEDD4L anomalies. MicroRNA-1(miR-1) regulates genes in the heart and skeletal muscle. Analysis of potential miR-1 target genes identified miR-1 as a direct *Nedd4* regulator; in addition, NEDD4L modulates cardiac development in *Drosophila* 69. Furthermore, miR-1-associated *Nedd4* regulation might help control the trafficking and catabolism of cardiac NEDD4L substrates in the cardiac tissue. However, further study is needed to clarify which substrate ubiquitinated by NEDD4L is involved in arrhythmia.

### The role of NEDD4L in cardiac regenerative repair

CircRNAs (circular RNAs) represent potent modulators of cardiac development and disease. Studies have found a new circRNA termed Nfix circRNA (circNfix). Down-regulation of circNfix can promote proliferation and angiogenesis in cardiomyocytes, inhibit the apoptosis of cardiomyocytes after myocardial infarction, alleviate cardiac insufficiency and improve patient prognosis. It was found that circNfix enhances Ybx1 (Y-box binding protein 1) interaction with NEDD4L, and induces Ybx1 degradation by ubiquitination via NEDD4L, which inhibits the expression of cyclin A2 and cyclin B1. In addition, suppression of superenhancer modulated circNfix promotes cardiac regeneration and enhances heart function following myocardial infarction via degradation of Ybx1, which may provide a promising strategy to improve prognosis after MI 70.

## ITCH

### ITCH function

The ubiquitin E3 ligase ITCH is a 113 kDa protein that contains N-terminal C2 (approximately 116 amino acids) domain, 2-4 protein-protein interaction WW (about 40 amino acid) which recognizes PPxY-(PY)-rich sequences, and C-terminal catalytic HECT domains 24, 71-72. The seven internal lysine moieties in ubiquitin may serve as chain extension sites 73. The polyubiquitin chain linked to lys48 is a proteolysis marker for 26S proteasome-induced degradation 74. ITCH is involved in multiple cell processes by targeting modulators of diverse pathways 75.

### The role of ITCH in myocardial infarction and cardiotoxicity

The occurrence of heart failure caused by MI or doxorubicin (DOX) is closely related to progressive left ventricular remodeling caused by oxidative stress 76. The main cause of excessive oxidative stress in HF cases is believed to be elevated intracellular reactivity related to the antioxidant defence against reactive oxygen free radicals (ROS) 77. Thioredoxin can be activated by inhibiting Thioredoxin Interacting Protein (TXNIP), which plays an important role in antagonizing ROS in cardiomyocytes 78. *Txnip* gene knockout in mice protects the animal's heart, and TXNIP degradation protects the heart of rats with ischemic heart disease 79. The WW domain recognizes a proxy PPxY sequence of TXNIP. The ITCH-dependent TXNIP UPS is essential for intracellular homeostasis 80. Overexpression of ITCH by enhancing degradation of TXNIP increases thioredoxin activity, thereby inhibiting the production of ROS, p38MAPK and p53, and suppressing the apoptosis of cardiomyocytes. Cytology confirmed that ITCH can maintain ROS homeostasis in cardiomyocytes 81. Meanwhile, cardiomyocyte-specific ITCH overexpression and transgenic mouse assays showed that ITCH plays a protective role in DOX and hydrogen peroxide-induced apoptosis and cardiotoxicity by degradation TXNIP. Therefore, ITCH plays a protective role in cardiotoxicity and provides a theoretical basis for MI-targeted therapy 82.

### The role of ITCH in diabetic cardiomyopathy

The main pathological changes of diabetic cardiomyopathy are cardiac hypertrophy, apoptosis and myocardial interstitial fibrosis 83. Calcium Sensitive Receptor (CaSR) belongs to the G protein-coupled receptor (GPCR) C family. Activation of CaSR increases intracellular Ca^2+^ concentration, upregulates ITCH, increases the ubiquitination level of SMAD7, and augments p-SMAD2 and p-SMAD3 amounts. Calhex 231, a CaSR inhibitor, suppresses the ITCH-ubiquitin proteasome and TGFβ1/SMADs pathway, thus inhibiting the proliferation of cardiac fibroblasts, reducing collagen deposition, and decreasing glucose-induced myocardial fibrosis. This regulatory pathway reveals a novel mechanism for ITCH in myocardial fibrosis, indicating that Calhex 231 may represent a novel drug for treating dilated cardiomyopathy 84.

## WWP1

### WWP1 function

WWP1 is also referred to as TIUL1 (TGIF interaction ubiquitin ligase 1) or AIP5 (atropine-1-interaction protein 5) 85. In humans, WWP1 comprises 922 amino acids, with a molecular weight of 110 kDa. WWP1 contains N-terminal C2, 4 tandem WW and C-terminal catalytic HECT domains. WW domains bind to precursor-rich peptide ligands that contain PPxY (PY), PPLP, PPR, and P(S/T) P motifs, respectively 86. WW domains 1 and 3 in WWP1 interact with the PY motif 87. The C-terminal has a HECT domain interacting with UbcH5 or UbcH7 (ubiquitin binding enzyme) and controls ubiquitin E3 ligase activity 88. HECT domain contains a catalytic cysteine with the capability of forming a covalent isopeptide bond with ubiquitin.

### The role of WWP1 in cardiac hypertrophy

WWP1 is found at high levels in the myocardium and skeletal muscle. Circular RNAs (circRNAs) may mediate the development of cardiac hypertrophy and the reprogramming of fetal genes 89. Total RNA from the left ventricular of mice with myocardial hypertrophy induced by isoprenaline hydrochloride was sequenced and the results were assessed by Gene Ontology and *Kyoto Encyclopedia analysis.* It was determined that ANF and mir-23a are the downstream targets of circRNA-WWP1, indicating that circRNA-WWP1 could inhibit cardiac hypertrophy by downregulating ANF and mir-23a, and that circRNA-WWP1 is a potential new therapeutic target for cardiac hypertrophy 90.

### The role of WWP1 in atrial fibrillation

Patients with atrial fibrillation (AF) have a high risk of cardiogenic stroke and further complications 91-92. Atrial fibrosis represents a critical regulator of integrity in atrial fibrillation, both structurally and functionally. Cardiac fibroblast proliferation is essential for atrial fibrosis and structural remodeling in individuals with atrial fibrillation 93-94. Transforming growth factor-B1 (TGF-b1) regulates cell proliferation in atrial fibrosis during AF. In addition, miR-21 suppresses endothelial progenitor cell proliferation via WWP1 targeting 95. WWP1 binds to Smad7 and enhances Smad7 interaction with TGF-b1 receptor, which is degraded 96. WWP-1 suppresses TGF-b-induced p-Smad2 97. Eventually, miR-21 suppresses cardiac fibroblast growth by inhibiting TGF-b1/Smad2 signaling through WWP1 upregulation 98.

## WWP2

### WWP2 function

WWP2 (about 870 amino acids) consists of N-terminal C2 domain, 4 tandem WW domain, and C-terminal HECT domain 99-100. WWP2 contains three subtypes, namely, the full-length (WWP2-FL, 870 amino acids), N-terminal subtype (WWP2-N, 336 amino acids), and C-terminal subtype (WWP2-C 440 amino acids) molecules. WWP2-N comprises the C2 and first WW (WW1) domains; WWP2-C encompasses the 4^th^ WW (WW4) and HECT domains, and WWP2-FL has all 3 domains 101. The HECT domain is structurally bilobal 102, and confers intrinsic ubiquitin ligase activity to WWP2. The WW domain of WWP2 recognizes the target protein through residues in the PY motif or substrate 103-104. WWP2 forms a self-inhibiting structure via intramolecular interactions of its C2 and HECT domains 105. WWP2 dose-dependently controls its ligase activity through polyubiquitination via K63 linkage 106.

### WWP2 function in myocardial fibrosis

The natural myocardial ischemia injury of dilated cardiomyopathy can cause myocardial fibrosis and eventually lead to heart failure 107-108. The development of tissue fibrosis involves the transforming growth factor β (TGFβ) superfamily of proteins 109. TGFβ1 binds directly to its receptor via the downstream effector protein SMADS 110. Through detailed genetic analysis of diabetic cardiomyopathy, WWP2 and *WWP2* mRNA expression in fibroid heart disease is only slightly increased 111. In primary cardiac fibroblasts, TGFβ1 stimulates the WWP2 N-terminal subtype to enter the nucleus, subsequently enhancing the activity of WWP2-FL to promote interaction with SMAD2, and promoting its mono-ubiquitination, which activates the downstream pro-fibrogenic gene program 112. Myocardial fibrosis is an important therapeutic target in HF cases 113, and WWP2 represents a regulator of TGFβ/SMAD signalling, which has critical functions in the activation and fibrosis of cardiac fibroblasts. Therefore, WWP2, as a new drug target, is of great significance to control pathological cardiac fibrosis and heart failure, and to improve the clinical prognosis of patients with heart disease. However, more studies are warranted to assess whether WWP2-mediated SMAD2 mono-ubiquitination interferes with or directly regulates SMAD2 complex activity 114, and to explain how WWP2 mono-ubiquitination affects SMAD2 nuclear output and regulation from a mechanical point of view 115.

### The role of WWP2 in cardiac remodeling

Cardiac remodeling, including cardiac hypertrophy and fibrosis, underlies HF development. Poly (ADP-Ribose) Polymerase 1 (PARP1) is an important damaging factor in cardiovascular disease, especially cardiac remodeling caused by various factors 77-79. In cardiac remodeling, cleaved-PARP1 amounts increase, which leads to extreme energy utilization by cardiomyocytes and cell injury 116-118. WWP2 mostly binds to the BRCT domain of PARP1, and can ubiquitinate its K249 and K418 residues. The ubiquitination level of PARP1 is decreased in MycCre+; WWP2^Fl/Fl^ mice, while the expression level of PARP1 induced by ISO is increased. Therefore, WWP2 can degrade PARP1 and protect from ISO-triggered cardiac remodeling. This provides a foundation for investigating novel approaches for the treatment of cardiac remodeling-associated disease 119.

### WWP2 function in vascular endothelial injury

Oxidative stress is important in the pathology of endothelial damage, initiating cardiovascular ailments, including atherosclerosis and hypertension 120-122. Endothelial/myeloid-specific *Wwp2* gene silencing markedly increases endothelial damage and vascular remodeling induced by angiotensin II. WWP2 promotes the degradation of the endothelial damage factor Septin4 via lysine residue 174 (K174), thus inhibiting formation of the Septin4-PARP1 endothelial damage complex 123. This study showed that the WWP2-septin4 pathway might represent a new target for preventing and treating atherosclerosis and hypertension. This was the first report to confirm WWP2 as the first NEDD4 family member to inhibit endothelial damage and vascular remodeling, providing an insight into IOS-related atherosclerosis and hypertension 124.

## SMURF1

### SMURF1 function

SMURF1 comprises N-terminal C2, tandem WW and C-terminal HECT domains 15. SMURF1 is localized intracellularly and recruited substrates, with the HECT domain containing the active site cysteine and interacting with E2 proteins, to form ubiquitin thioesters and promote substrate ubiquitination.

### SMURF1's role in pulmonary hypertension

Pulmonary hypertension (PAH) is characterized by thickening of the middle layer of the pulmonary artery, eventually causing HF and death 125-126. PAH is related to bone morphogenetic protein receptor 2 (*BMPR2*) gene mutation 127. When bone morphogenesis proteins interact with BMPR2, the cytoplasmic proteins R-SMAD1/5/8 are phosphorylated. R-SMADs interact with the cofactor SMAD4 and undergo nuclear translocation, further regulating cell growth, proliferation and differentiation 127. The degradation of R-SMADs by SMURF1 blocks this pathway 128. Damage to the BMPR2 pathway leads to pulmonary artery occlusion and right ventricle afterload increase 129. MiR-424(322), as a SMURF1 target can maintain the signal transduction of BMPR2. However, hypoxia-induced miR-424(322) secretion results in reduced SMURF1 expression, increased right ventricular hypertrophy and heart failure. In PAH rats, the level of miR-424(322) shows a negative correlation with SMURF1 amounts in the hypertrophic right ventricle. Therefore, the miR-424(322)-SMURF1 regulatory pathway could be explored for diagnosing pulmonary hypertension by measuring peripheral blood miR-424(322) 130.

Another study revealed that mir-140-5p with the target of *SMURF1* mRNA, are decreased in individuals with PAH. Indeed, mir-140-5p and *SMURF1* amounts are negatively correlated. As a BMPR2 signaling suppressor, *Smurf1* knockout mice show right ventricular hypertrophy and decreased pulmonary microvascular remodeling. As mir-140-5p is cytotoxic, a SMURF1 inhibitor for the treatment of PAH, could replace mir-140-5p for treating PAH by inhibiting the degradation of BMPR2 and reducing the therapeutic toxicity 131-132.

### The role of SMURF1 in angiogenesis

Angiogenesis represents a multi-step event that involves endothelial cell activation, proliferation and migration. TGFβ family members have critical functions in the development of the vascular system 133. SMASD7 and SMURF1 exert negative effects on TGFβ1-induced VEGF expression and SMAD3/4-mediated VEGF expression. VEGF is effective for treating pathological angiogenesis, therefore, SMURF1 has the function of regulating angiogenesis 134. Receptor-Like Kinase 1 (ALK1) is necessary for vascular development, remodeling and abnormal angiogenesis 135. Bone morphogenic 9 (BMP9) and BMP10 specifically activate endothelial cells 136. Human umbilical vein endothelial cell (HUVEC) treatment with metformin and AMPK inducers activate AMPK, increasing the expression of SMURF1, which leads to ALK1 degradation and the inhibition of BMP9-induced SMAD1/5 phosphorylation and angiogenesis. The role of SMURF1 in this regulatory pathway indicate a new clinical application of AMPK activators based on metformin and other drugs, suggesting a combination with other strategies to improve therapeutic effects in anti-VEGF resistance-related disease 137.

### The role of SMURF1 in heart failure

With the aging of the world population, HF incidence is increasing 138. Activin A (ActRI), a ligand of ActRII, is associated with aging and muscle atrophy in several disease 139. The expression of ActRI signaling in the heart is increased, and heart function is damaged with aging. Moreover, SMURF1 is considered a key downstream effector of the ActRI signaling pathway, which promotes the degradation of sarcoplasmic Ca^2+^ ATPase (SERCA2a), a key factor determining the function of cardiomyocytes. The catabolic pathway mediated by SMURF1 inhibits ActRI signaling, thus can serve for targeted treatment of HF 140.

### The role of SMURF1 in myocardial fibrosis

The therapeutic potential of endothelial colony forming cells (ECFCs) in the ischemic environment may be impaired. Normoxia significantly improves the activation of cultured cardiac fibroblasts. These effects were weakened in the hypoxia group. Mir-10b-5p was enriched in normoxia, but not in hypoxia. Mir-10b-5p inhibits the expression of *Smurf1* mRNA. Therefore, *Smurf1* downregulation by mir-10b-5p could blunt the anti-fibrotic effect of hypoxia, and SMURF1 has a potential function in alleviating myocardial fibrosis 141. However, it needs to make a profound study how SMURF 1 participates in myocardial fibrosis through ubiquitination of downstream substrates.

### The role of SMURF1 in heart valve disease

Heart valve disease represents a main mortality factor and morbidity. Epithelial-to-mesenchymal transition (EMT) of the inner membrane cell subpopulation in the atrioventricular septum (AVC) represents a key step of heart valve development 142. In accordance with the activation of PAR6/SMURF1 signaling downstream of TGFβR3, targeting ALK5, PAR6 or SMURF1 markedly inhibits the EMT response to TGFβ2 or BMP2. The need for ALK5 activity, PAR6, and SMURF1 in TGFβR3-associated EMT of endocardial cells corroborates the pathway's involvement with tight junctions. Therefore, SMURF1 is involved in heart valve disease 143.

### The role of SMURF1 in Heart Development

Heart development comprises complex developmental events, involving the deployment of multiple cell lines 144. The proepicardium (PE) represents an important transitory cauliflower-like structure between the heart and liver primordia. MicroRNAs highly contribute to the development of the cardiovascular system. It was found that mir-125, miR-146, miR-195 and mir-223 specifically promote chicken cardiac myogenesis of PE/ST explants and also contribute to embryonic epicardium, a process controlled by Smurf1- and Foxp1 145.

Additionally, heart SMURF1 amounts are high in mice during development 146, mainly distributing in ectodermal tissues. SMURF1 affects many processes in the developing heart, e.g., OFT septation and SMC generation in the main and coronary arteries. Smurf1 plays a role in CNC-mediated OFT septation by regulating the steady state levels of BMP and TGFβ signaling effectors to induce EndoMT, CNC delamination and migration 147-148. In addition, SMURF1 highly contributes to SMC differentiation from the epicardium by regulating BMP-dependent SMAD signaling, together with RHOA, PAR6 and TGFβ-RIII18 149. Furthermore, SMURF1 exerts effects at the cilium to control baseline SMAD1/5 activation to regulate BMP signaling in the generation of cardiomyocytes, substantially contributing to cell-type specification 147. The mechanisms of SMURF1 participation in heart development have provided insights for improving *in vitro* differentiation of cardiomyocytes to treat cardiovascular disorders.

## SMURF2

### SMURF2 function

SMURF2 contains an N-terminal C2 150, two or more central WW domains interacting with the PY motif of the target protein or the adaptor protein, and a HECT domain at the C-terminus. SMURF2 receives ubiquitin from E2 at its cysteine 716 (Cys 716) active site and transfers it to the target protein to control its stability, localization and function 150. SMURFs were originally considered negative regulators of TGFβ/BMP signaling. Through interaction with PY motifs in most R-SMADs and SMAD inhibitors, SMURFs regulate SMADs or TGFβ/BMPs, or other downstream components of the targeted pathway 128, 151-152. After TGFβ-induced expression, the inhibitor of the adaptor protein SMAD7 forms a complex with SMURF2 in the nucleus 126. SMURF2 then down-regulates TGFβ signal transduction by targeting itself, SMAD7 and TGFβ receptor kinase. In the presence of active TGFβ signaling, SMAD2 interacts with the tryptophan-rich WW domain of SMURF2 through the proline-rich PPxY motif 153.

### The role of SMURF2 in calcified aortic valve disease

Calcified aortic valve disease incidence is very high in elderly individuals. In humans, TGFβ1 and BMP2 promote the pre-osteogenic induction of aortic valve interstitial cells (AVICS), which have a critical function in valve calcification 154-155. TGFβ1 and BMP2 upregulate mir-486 and downregulate mir-204 for promoting osteogenic activity, while mir-486 downregulates *Smurf2* to enhance mir-204 downregulation. *Smurf2* gene knockout enhances TGFβ1 or BMP2-induced downregulation of mir-204 and results in elevated amounts of the osteoblast markers OSX and RUNX2. Therefore, the mir-486-SMURF2-SMAD regulatory pathway has a critical function in modulating the osteogenic effects of TGFβ1 and BMP2. Targeting this modulatory circuit might have therapeutic potential in inhibiting aortic valve calcification 156.

### The role of SMURF2 in myxomatous mitral disease (MMVD)

Myxomatous mitral disease (MMVD) represents a commonly detected heart disease 157. Increased phosphorylation of SMAD2/3 158 and *SMURF2* expression indicate that TGFβ1 signaling occurs through a typical signaling cascade. The levels of CTGF, MMP2, NOX2 and NOX4 159-160 in mitral valves of SOD1-deficient mice were shown to be significantly increased. In addition, treatment of mouse valve stromal cells with cell permeability antioxidants resulted in reduced expression of TGFβ1-induced SMURF2 involved in promoting fibrosis and matrix remodeling genes. The study of this regulatory pathway has potential guidance for targeted therapy of MMVD.

### The role of SMURF2 in vascular smooth muscle proliferation

Platelet-derived growth factor BB (PDGF-BB) and TGFβ1 are important growth factors regulating vascular smooth muscle cell (VSMC) proliferation. PDGF-BB increases the levels of ALK5, SMURF2, pSMAD2/3 and SMAD4, but decreases those of SMAD2 and SMAD7; these changes are partially reversed by neutralizing anti-TGFβ1 antibodies. The TGFβ signaling pathway mediates PDGF-BB effect in VSMCs by regulating downstream proteins such as SMURF2 161. This study revealed the molecular mechanism of SMURF2 in vascular smooth muscle and laid a theoretical foundation for the study of vascular disease.

### The role of SMURF2 in Vascular endothelial injury

ROS are important signaling molecules that maintain the homeostasis of the cardiovascular system 162. However, significant increases in ROS levels are closely related to the occurrence and development of cardiovascular ailments, including HF 163, atherosclerosis 164, hypertension 165 and ischemic heart disease 166, resulting in inflammation, fibrosis and apoptosis 167-168. Oxidative stress-associated injury is closely related to ubiquitination. In the cardiovascular system, activation of PARP1 could cause vascular endothelial cell damage 168. Decreasing PARP1 activity could reduce the development of atherosclerotic plaques 169. The interaction between SMURF2 and PARP1 is enhanced under oxidative stress. In H2O2-treated human umbilical vein endothelial cells, SMURF2 was shown to degrade PARP1 to reduce ROS production and apoptosis, thereby alleviating oxidative stress injury 170. The molecular mechanism of SMURF2 in atherosclerosis provides an insight into targeted treatment of the disease.

### The role of SMURF2 in ischemia-reperfusion

I/R represents a commonly diagnosed and lethal ailment. Studies found that overexpression of mir-322/503 can enhance the expression of p-Akt and p-GSK3β, reduce infarct area, inhibit apoptosis and promote cell proliferation. It was also found that mir-322/503 could directly bind to Smurf2 gene for downregulation at the translational level, reducing the ubiquitination and degradation of EZH2 by Smurf2, activating Akt/GSK3 β signal transduction and protecting cells from I/R injury 171.

### The role of SMURF2 in myocardial fibrosis

After myocardial infarction, the heart is severely remolded, and a structured collagen network is generated to resist circumferential deformation 172 for preventing scar rupture and promoting survival. However, abnormal biosynthesis of matrix proteins eventually results in cardiac fibrosis and heart failure 173. TGF-β plays a role in heart failure development by promoting cardiac fibrosis 174. Smurf2 is considered a critical TGF-β pathway suppressor via Smad2 degradation 175. In addition, Smurf2 and Smad7 jointly repress TGF-β signaling 176. Smurf2's function is therefore paradoxical. This contradictory behavior of Smurf2 is because nuclear Smurf2 induces the TGF-β pathway, while cytosolic Smurf2 exerts opposite effects. As cardiac fibrosis appears to show altered TGF-β signaling, investigating Smad-regulating proteins may help identify an effective way to treat myocardial fibrosis 177.

## Conclusions and Prospects

A major HECT group of ubiquitin ligases is the NEDD4 family 5, 6. NEDD4 proteins identify different substrate proteins to perform ubiquitination catalysis, thus participating in various cardiovascular diseases. The present review investigated the functions and regulation of the NEDD4 family in association with cardiovascular disease **(Table 1).**

**Figure 2:**
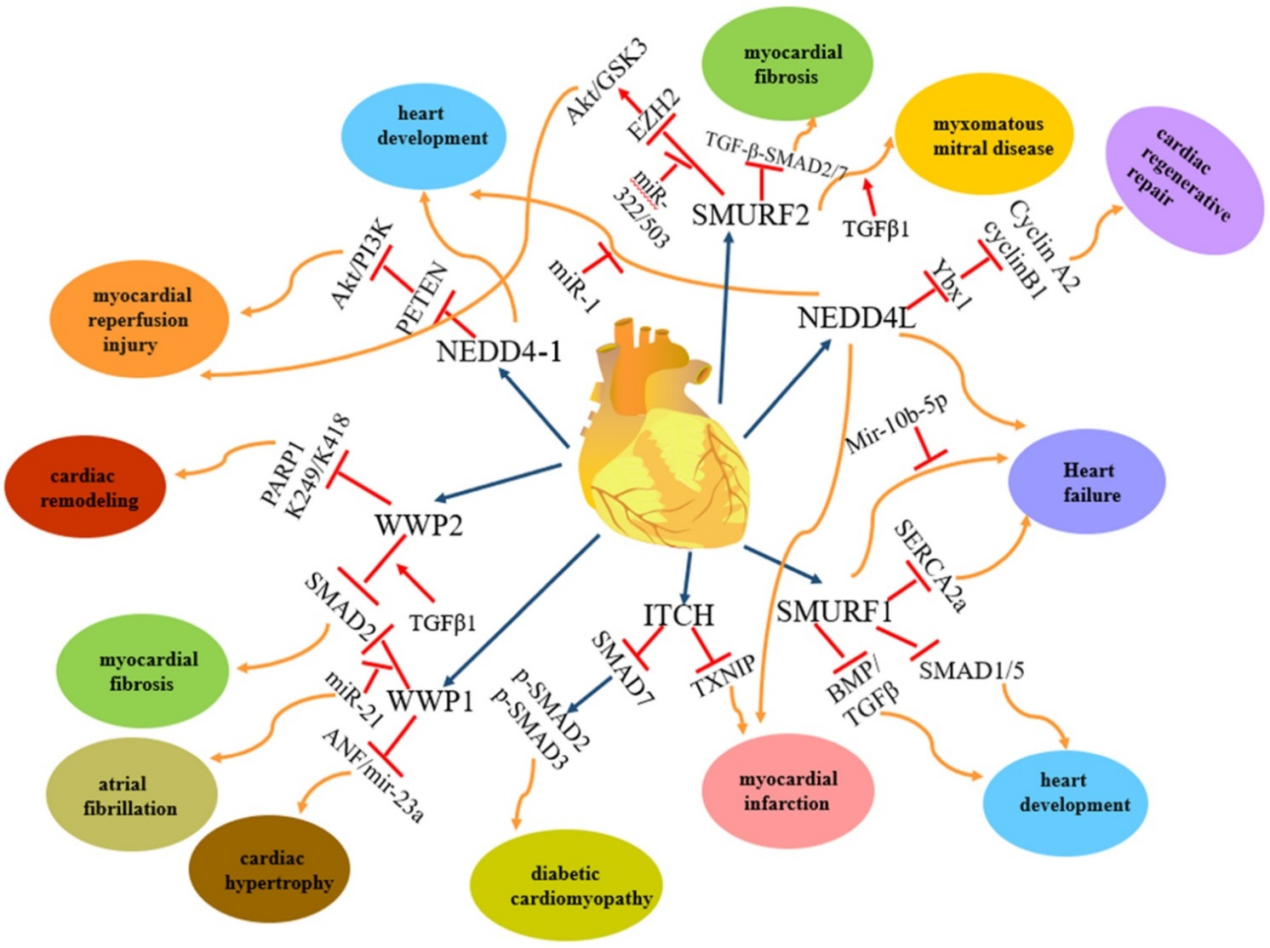
** The regulation and functions of NEDD4 E3 ligases in cardiac disease.** The regulatory pathway of NEDD4 E3 ligases including NEDD4-1, NEDD4L, ITCH, WWP1, WWP2, Smurf1 and Smurf2 in cardiac disease, such as myocardial reperfusion injury, heart development, heart failure, myocardial infarction, diabetic cardiomyopathy, cardiac hypertrophy, myocardial fibrosis, cardiac remodelling, myxomatous mitral disease.

**Figure 3:**
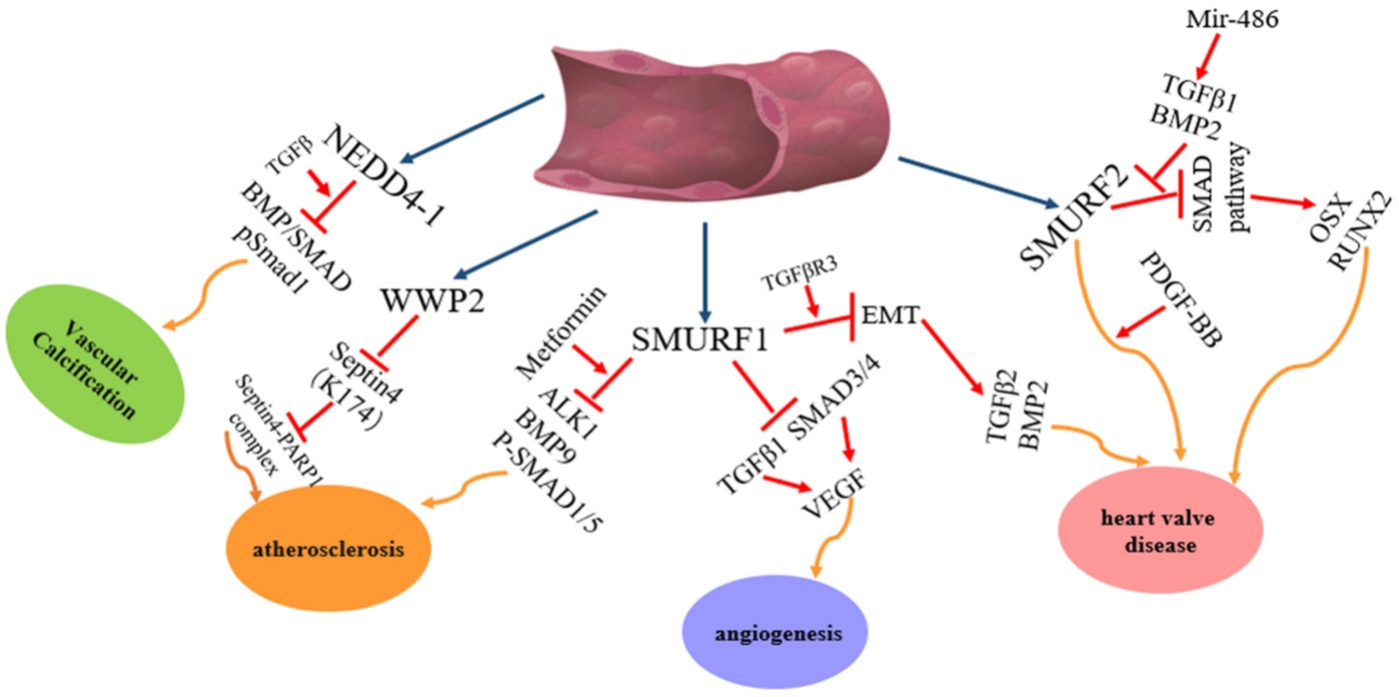
** The regulation and functions of NEDD4 E3 ligases in vascular disease.** The regulatory pathway of NEDD4 E3 ligases including WWP2, Smurf1 and Smurf2 in vascular disease, such atherosclerosis, angiogenesis, heart valve disease.

Among the nine members of the NEDD4 family, NEDD4-1, NEDD4L, ITCH, WWP1, WWP2, SMURF1 and SMURF2 are involved in heart diseases. However, each of them plays different roles in heart diseases **(Figure 2)**: NEDD4-1 participates in myocardial ischemia-reperfusion injury 43 and abnormality of heart development 44; NEDD4L and SMURF1 are involved in heart failure 68, myocardial infarction 68 and heart development 69, 139-141, 147-149; WWP1 participates in cardiac hypertrophy 89-90 and atrial fibrillation 95-98; WWP2 is involved in myocardial fibrosis 110-115 and cardiac remodelling 116-119; ITCH participates in myocardial infarction 78-82 and diabetic cardiomyopathy 83-84 and SMURF2 participates in myocardial fibrosis 172-177 and myocardial ischemia-reperfusion injury 171. In addition, NEDD4-1, NEDD4L, WWP2, SMURF1 and SMURF2 are involved in vascular disease **(Figure 3).** NEDD4-1 participates in vascular calcification 45-48 and NEDD4L participates in hypertension 22, 56-67. WWP2 is involved in vascular endothelial injury 123-124, SMURF1 in atherosclerosis 135-137 and pulmonary hypertension 127-132, and SMURF2 in myxomatous mitral disease 158-160 and vascular endothelial injury 168-170. However, whether NEDL1 and NEDL2 play biological functions in cardiovascular related disease needs further study.

The research indicated that suppression of circNfix can promote cardiac regeneration and enhance heart function following myocardial infarction via degradation of Ybx1 by NEDD4L, which may provide a promising strategy to improve prognosis after MI 70. It reveals the role that ITCH/ TGFβ1/SMADs pathway inhibits myocardial fibrosis, indicating that Calhex 231 may represent a novel drug for treating dilated cardiomyopathy 84. It was indicated that circRNA-WWP1 could inhibit cardiac hypertrophy by downregulating ANF and mir-23a by Gene Ontology and Kyoto Encyclopedia analysis, and that circRNA-WWP1 is a potential new therapeutic target for cardiac hypertrophy 90. Metformin and AMPK activators activate AMPK and SMURF1, subsequently, degradation of ALK1, and inhibition of BMP9-induced SMAD1/5. It indicates a new clinical application of AMPK activators combination with metformin to improve therapeutic effects in anti-VEGF resistance-related disease 137. The mir-486-SMURF2-SMAD regulatory pathway in modulating the osteogenic effects might have therapeutic potential in inhibiting aortic valve calcification 156. Thus, it will play an important role in revealing multiple various regulatory mechanisms and functions of NEDD4 E3 ligase family to explore the above issues deeply, and provide new insights for the development of cardiovascular disease.

## Figures and Tables

**Table 1:** The NEDD4 family functions and regulation

Enzymes	Mechanisms	Biological response	References
NEDD4-1 (RPF1)	Activate Akt/PI3K signaling pathway and degradate PTEN by binding to its N-terminal	myocardial reperfusion injury (I/R)	40-42
	NEDD4-1 KO mice show a double outlet right ventricle, an endocardial cushion defect and abnormalityof the cephalic plexus vein of embryos	abnormality of heart development	43,44
	NEDD4-1 deletion in mouse tissues showed deformed aortic structures. Suppress BMP/Smad pathway via degradation of C-terminal pSmad1 activated by TGF-β.	Vascular Calcification	45-48
NEDD4L (NEDD4-like or NEDD4-2)	WW domain of NEDD4L recognizes β- and γ-subunits of PY motif of ENaC in C domain	hypertension	22,56-60
	WNK1 activates SGK1 phosphorylating NEDD4L on serine 444 by interaction with the chaperone 14-3-3 and results in a reduction of ENaC ubiquitination. IL17A increased NCC activity in an SGK1/NEDD4L dependent pathway.	hypertension	61-67
	downregulate Nav1.5 by ubiquitination; defect the NEDD4L C2 isoform	heart failure (HF); myocardial infarction (MI)	68
	miR-1 regulates the 3′-UTR of Nedd4L	heart development	69
	circnfix enhanced the interaction between Ybx1 and NEDD4L, and induced Ybx1 degradation by NEDD4L, which inhibited cyclin A2 and cyclin B1.	cardiac regenerative repair	70
ITCH (AIP4)	TXNIP binds to HECT domain of ITCH and WW domain recognizes PPxY motif of TXNIP.	myocardial infarction (MI) or doxorubicin (DOX)	78-82
	ITCH which is upregulated by increasing intracellular Ca^2+^ via CaSR, increases the ubiquitination level of SMAD7, and upregulates the levels of p-SMAD2 and p-SMAD3.	diabetic cardiomyopathy	83-84
WWP1 (TIUL1 or AIP5)	circRNA-WWP1 down-regulates ANF and mir-23a	cardiac hypertrophy	89-90
	miR-21 inhibits cardiac fibroblasts proliferation by inactivating the TGF-b1/Smad2 via up-regulation of WWP1	atrial fibrillation	95-98
WWP2 (AIP2)	TGFβ1 stimulates the WWP2 N-terminal subtype to enter the nucleus. The WWP2-N subtype enhances the activity of WWP2-FL to promote interaction with SMAD2, and promote its monoubiquitination.	myocardial fibrosis	110-115
	WWP2 interacts with PARP1 in its BRCT domain and ubiquitinate K249 and K418 of PARP1.	cardiac remodelling	116-119
	WWP2 promoted the degradation of Septin4-K174, thus inhibiting formation of the Septin4-PARP1 complex.	Vascular endothelial injury	123-124
SMURF1	Secretion of miR-424 (322) by PAECs results in down-regulation of SMURF1, thus blocking the degradation of R-SMADs and increasing BMPR2 pathway activity.	pulmonary hypertension	127-130
	The binding target of mir-140-5p was SMURF1 mRNA. A SMURF1 inhibitor can replace mir-140-5p by inhibiting ubiquitination of BMPR2.	pulmonary hypertension	131-132
	SMURF1 have negative effects on TGFβ1-induced VEGF expression and SMAD3/4-mediated VEGF expression.	angiogenesis	134
	Metformin and AMPK activators activate AMPK and SMURF1 is up-regulated, leading to the degradation of ALK1, and inhibition of BMP9-induced SMAD1/5 phosphorylation and angiogenesis.	angiogenesis	135-137
	SMURF1 promotes degradation of (SERCA2a) therefore inhibit actri signalling.	heart failure	139-140
	Mir-10b-5p inhibits the expression of Smurf1	heart failure	141
	PAR6/SMURF1 pathway downstream of TGFβR3 actives, targeting ALK5, PAR6 or SMURF1 significantly inhibited the EMT response to TGFβ2 or BMP2.	heart valve disease	143
	SMURF1 regulates BMP and TGFβ signaling to induct EndoMT in CNC-mediated OFT septation, CNC delamination, migration and in SMC differentiation from the epicardium, together with RHOA, PAR6 and TGFβ-RIII18. SMURF1 modulates SMAD1/5 activation to regulate BMP signaling during cardiomyogenesis at the cilium toand.	Heart Development	147-149
SMURF2	Mir-486, up-regulated by TGFβ1 and BMP2, inhibits SMURF2-SMAD regulatory pathway leading to enhance mir-204 down regulation and results in increased of OSX and RUNX2.	valve disease	154-156
	TGFβ1 signalling increases phosphorylation of SMAD2/3 and SMURF2 expression leading to promote fibrosis and matrix remodelling genes.	myxomatous mitral disease	158-160
	PDGF-BB increases expression of ALK5, SMURF2, pSMAD2/3 and SMAD4, but decreases expression of SMAD2 and SMAD7	vascular smooth muscle proliferation	161
	SMURF2 degrades PARP1 through the ubiquitin proteasome pathway	Vascular endothelial injury	168-170
	mir-322 / 503 binds to Smurf2 gene and inhibits its translation to reduce the degradation of EZH2, to activate Akt/GSK3 β signal transduction and protect cells from I/R injury	ischemia-reperfusion	171
	Smurf2 inhibits TGF-β pathway via degradation of Smad2. In addition, Smurf2 conjunction with Smad7 to halt TGF-β signal transduction.	myocardial fibrosis	172-177
NEDL1 (HECW1)	None	None	None
NEDL2 (HECW2)	None	None	None
